# Development of prediction models of spontaneous ureteral stone passage through machine learning: Comparison with conventional statistical analysis

**DOI:** 10.1371/journal.pone.0260517

**Published:** 2021-12-01

**Authors:** Jee Soo Park, Dong Wook Kim, Dongu Lee, Taeju Lee, Kyo Chul Koo, Woong Kyu Han, Byung Ha Chung, Kwang Suk Lee

**Affiliations:** 1 Department of Urology, Yonsei University College of Medicine, Seoul, Korea; 2 Department of Urology, Sorokdo National Hospital, Goheung, Korea; 3 Department of Oral and Maxillofacial Surgery, Yonsei University College of Dentistry, Seoul, Korea; 4 Department of Mechanical Engineering, Yonsei University College of Engineering, Seoul, Korea; Istanbul Universitesi, TURKEY

## Abstract

**Objectives:**

To develop a prediction model of spontaneous ureteral stone passage (SSP) using machine learning and logistic regression and compare the performance of the two models. Indications for management of ureteral stones are unclear, and the clinician determines whether to wait for SSP or perform active treatment, especially in well-controlled patients, to avoid unwanted complications. Therefore, suggesting the possibility of SSP would help make a clinical decision regarding ureteral stones.

**Methods:**

Patients diagnosed with unilateral ureteral stones at our emergency department between August 2014 and September 2018 were included and underwent non-contrast-enhanced computed tomography 4 weeks from the first stone episode. Predictors of SSP were applied to build and validate the prediction model using multilayer perceptron (MLP) with the Keras framework.

**Results:**

Of 833 patients, SSP was observed in 606 (72.7%). SSP rates were 68.2% and 75.6% for stone sizes 5–10 mm and <5 mm, respectively. Stone opacity, location, and whether it was the first ureteral stone episode were significant predictors of SSP. Areas under the curve (AUCs) for receiver operating characteristic (ROC) curves for MLP, and logistic regression were 0.859 and 0.847, respectively, for stones <5 mm, and 0.881 and 0.817, respectively, for 5–10 mm stones.

**Conclusion:**

SSP prediction models were developed in patients with well-controlled unilateral ureteral stones; the performance of the models was good, especially in identifying SSP for 5–10-mm ureteral stones without definite treatment guidelines. To further improve the performance of these models, future studies should focus on using machine learning techniques in image analysis.

## Introduction

Ureteral stones are the most common urologic emergency. They are associated with severe pain, renal obstruction, and urinary tract infection [1]. Indications for the management of ureteral stones are not clearly defined. The clinician’s choice determines whether to wait for spontaneous ureteral stone passage (SSP) or perform active treatment, including extracorporeal shock wave lithotripsy (ESWL), ureteroscopy, laparoscopic removal, or percutaneous treatment. However, in some instances, active treatment may be provided without waiting for SSP, in well-controlled patients, to avoid unwanted complications, including recurrent attacks of renal colic, urinary tract infection, and deterioration of renal function, which might be considered as over-treatment. Therefore, suggesting the possibility of SSP would help make a clinical decision regarding ureteral stones.

Predictive modelling of patient outcomes involves development of mathematical models to predict individual patient outcomes [2]. Models can be based on traditional statistical techniques or artificial intelligence (AI) techniques [2]. AI approaches are capable in not only processing imprecise and uncertain data which is common in clinical and biological data, but also in processing big data that are too large or complex for conventional statistical techniques [3]. In the fields of ureteral stones, AI has been used in predicting stone-free rate, however, no study has been performed for usage of AI in predicting SSP [2].

Intervention for ureterolithiasis is recommended after a 4-week observation period for patients with a ureteral stone who visit the outpatient department through the emergency department. As recommended by the European Association of Urology (EAU) and American Association of Urology (AUA) joint guidelines [4], our team determines the SSP rate of ureteral stones among patients with pain control and without complications during the observation period. Therefore, this study aimed to identify predictive prognostic factors for SSP. In addition, using those factors, we developed and compared prediction models of SSP using machine learning and logistic regression.

## Materials and methods

The Institutional Review Board of the Yonsei University Health system (project no: 2019-0959-001) approved the study. The data were analyzed anonymously in retrospective manners, so the consent was waived. Please contact the corresponding author or the Institutional Review Board of the Yonsei University Health system (irb@yuhs.ac) for the request for original data that has been used for this analysis. Upon request by the researchers for original data for this study, the Institutional Review Board of the Yonsei University Health system will review whether researchers meet the criteria for access to confidential data.

### Study population

After institutional review board approval, a retrospective review was performed on patients who were referred to the urology outpatient department and diagnosed with unilateral ureteral stones at our emergency department between August 2014 and September 2018. Of the initial 868 patients, 35 were excluded for the following reasons: (I) 25 had uncontrolled pain, (II) four experienced spiking fever owing to renal obstruction, and (III) six were employed as airline pilots or soldiers, in whom an episode of intractable renal colic could be dangerous.

### Institutional protocol for patients with ureteral stone episode

According to the renal colic management protocol of our emergency department, all patients underwent a detailed medical evaluation, including history taking; physical examination; urinalysis; complete blood count; routine serum chemistry measurements; kidney, ureter, and bladder radiography (KUB); and non-contrast-enhanced computed tomography (NCCT). Fluoroscopy or ultrasound has not been done.

In the urology outpatient department, patients were questioned about pain severity, complications, ureteral stone history, and sensation or observation of stone fragments during urination. Fluid intake of >2 L per day was recommended during the observation period. We performed NCCT 4 weeks from the first stone episode if the stone was not spontaneously expelled. For patients who did not experience SSP, the decision to continue follow-up after 2 weeks or perform intervention was based on the physician’s discretion and patient preference.

### Definition of variables related to stone characteristics

The diagnosis of ureteral stones was based on the presence of an unequivocal finding of a stone on NCCT. Stone size was defined according to its largest diameter and was stratified into groups: those measuring <5 mm, 5–10 mm, and >10 mm. The location of the stone was classified into two groups based on its anatomical position in the upper or lower ureter. Plain radiographic characteristics were used to classify stones as radiopaque or radiolucent [4]. In patients with a history of ureteral stones, the present episode on the other side was considered the first ureteral stone. The estimated glomerular filtration rate (eGFR) was determined using the Modification of Diet in Renal Disease formula [5].

### Assessment of predicting stone spontaneous passing at 4 weeks from the stone episode

Predictors of SSP were analyzed based on 1) laboratory investigations, including urinalysis, complete blood count, and routine serum chemistry measurements; 2) radiographic results, such as stone size, stone location, radiographic characteristics, and presence of hydronephrosis; 3) medical expulsive therapy (MET) using α-blockers; and 4) a history of stones and interventions.

### Machine learning

Prior to formulating machine learning models, the data set was randomly divided into two mutually exclusive sets for random allocation of SSP and non-SSP: training (80%) and validation (20%) [6]. The training set was used to construct the prediction model, while the validation set was used for validating the performance of each model. For the training set, the set was randomly divided into two sets: training (80%) and testing (20%). A concise description of each machine learning algorithm is provided below. All machine learning models were implemented using the Keras framework with R programming language (R Core Team, Vienna, Austria, 2016) [7].

### Multilayer perceptron (MLP)

MLP is a class of artificial neural network (ANN) with multiple or deep layers of nodes. Each node is a neuron resembling the connectivity patterns of that of animals, and uses a non-linear activation function that enables distinguishing linearly inseparable data. The Keras framework, a recent deep learning interface, was employed to construct an MLP model in this study [7].

**Fig 1:**
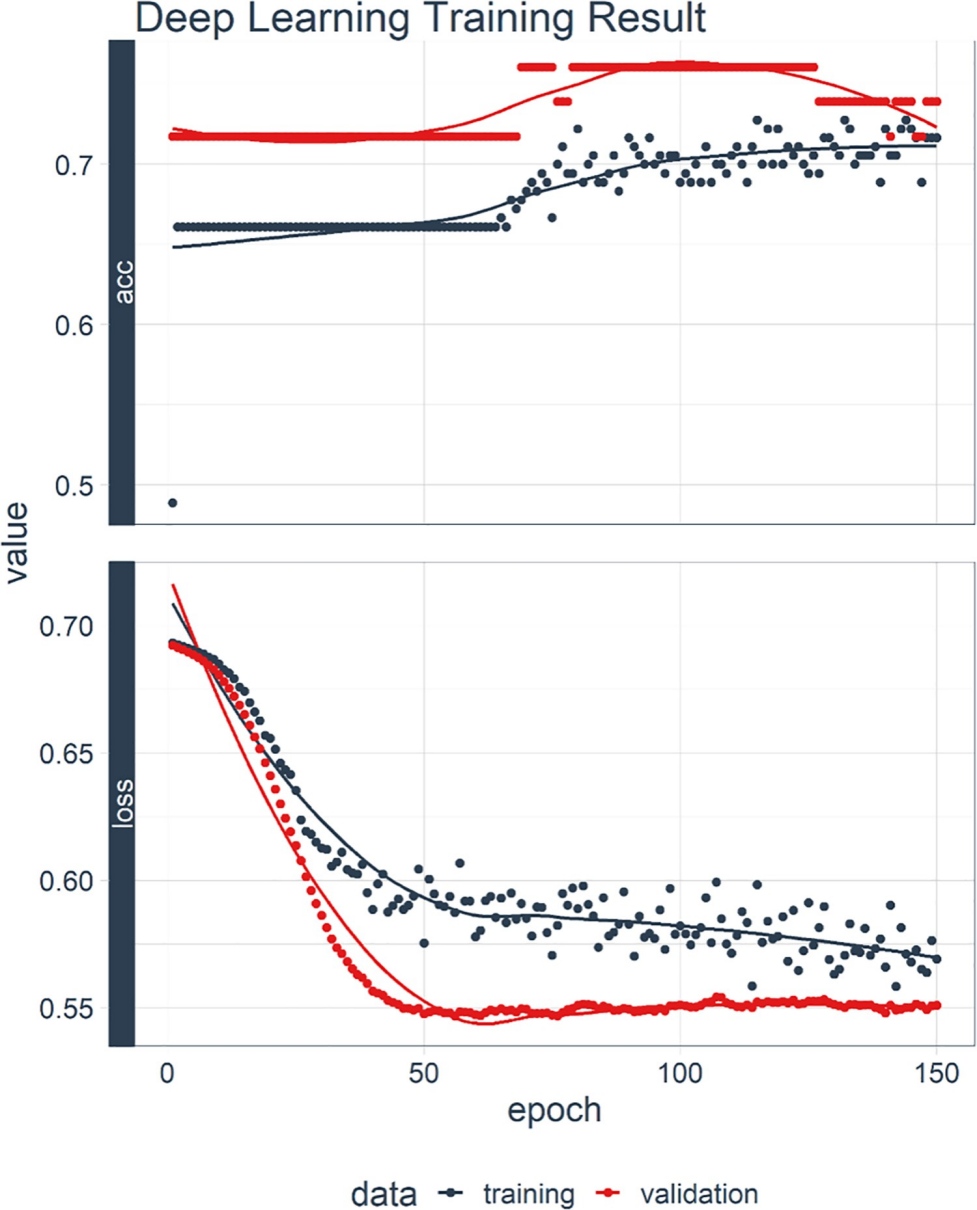
Training and validation curves. The progress of training is visualized by plotting the loss of each iteration. The accuracy of the validation set is also plotted together for every epoch.

The architecture of the MLP model used in this study was composed of an input layer, three fully connected hidden layers with 128 and 16 nodes, and an output layer. The input layer referred to input data, such as predictors of SSP. The hidden layers were layers where computing of the input features occurred. The node in the output layer represented the computed prediction [8]. The learning process of this MLP is visualized in Fig 1.

### Statistical analysis

Results were reported as mean ± standard deviation for continuous variables and as percentages for categorical variables. For univariate analyses, the *t*-test was used for continuous variables and the chi-square test or Fisher’s exact test for categorical variables. Univariate logistic regression analyses were performed, and multivariate logistic regression analyses were performed on significant factors associated with SSP in the univariate analyses. The area under the receiver operating characteristic (ROC) curve (AUC) was used to compare the performance of models. All reported p-values were two-sided, and statistical significance was set at p<0.05. Statistical analyses were performed using the Statistical Package for Social Sciences, version 23.0, for Windows (SPSS, Chicago, IL, USA).

## Results

Patient characteristics are shown in Table 1. Of the 833 patients included for analysis, SSP was observed in 606 (72.7%). eGFR, the first episode of ureteral stone, the spontaneous passage of the previous stone, location, opacity of the stone, and the presence of hydronephrosis were significantly different between the groups with and without SSP.

**Table 1:** Comparison of patients according to the spontaneous ureteral stone passage (SSP).

	SSP (-)	SSP (+)	P-value
No. of patients (%)	227 (27.3)	606 (72.7)	
Sex (male), n (%)	157 (69.2)	403 (66.5)	0.467
Age (yrs)	45.7 ± 14.3	47.9 ± 14.3	0.051
BMI (kg/cm^2^)	24.1 ± 3.6	24.3 ± 3.7	0.532
eGFR (ml/min/1.73 m^2^)	97.5 ± 73.5	89.0 ± 24.7	0.014
First ureteral stone episode (yes), n (%)	104 (45.8)	419 (69.1)	<0.001
Previous SSP (yes), n (%)	8 (3.5)	43 (7.1)	0.027
Side (right), n (%)	114 (50.2)	300 (49.5)	0.854
Location (lower), n (%)	115 (50.7)	434 (71.6)	<0.001
Stone size (mm)	4.99 ± 2.67	4.52 ± 1.92	0.018
<5 mm, n (%)	127 (55.9)	394 (65.0)	0.052
5–10 mm, n (%)	93 (41.0)	199 (32.8)	
≥10 mm, n (%)	7 (3.1)	13 (2.2)	
α-blocker usage (yes), n (%)	38 (16.7)	77 (12.7)	0.155
Presence of hydronephrosis (yes), n (%)	224 (98.7)	580 (95.7)	0.008
Radiopaque (yes), n (%)	90 (39.6)	344 (56.8)	<0.001
Multiple stones (yes), n (%)	4 (1.8)	10 (1.7)	0.999
RBC count in urine (/μL)	2171.7 ± 5208.01	2150.16 ± 5864.09	0.962
WBC count in urine (/μL)	37.77 ± 163.62	39.26 ± 279.98	0.940

Data are shown as mean ± SD or number of subjects (%).

BMI, body mass index; eGFR, estimated glomerular filtration rate; RBC, red blood cell count; SSP, spontaneous ureteral stone passage; WBC, white blood cell count.

P-value calculated using *t*-test for continuous variables and chi-square test or Fisher’s exact test for categorical variables.

In the multivariate analysis for predicting SSP, first ureteral stone episode (Odds ratio (OR) = 2.53 (1.819–3.510), p<0.001), location of stone (lower) (OR = 2.43 (1.738–3.390), p<0.001), and opacity of the stones (radiopaque) (OR = 1.91 (1.36–2.655), p<0.001) were identified as independent predictors of SSP (Table 2). The stone size and α-blocker usage (MET) were not identified as significant predictors of SSP.

**Table 2:** Univariate and multivariate logistic regression analyses of the factors predicting the spontaneous passage of ureteral stones.

	*Univariate*		*Multivariate*	
	Odds Ratio (95% CI)	P-value	Odds Ratio (95% CI)	P-value
Sex (male)	0.89 (0.637–1.229)	0.446		
Age (yrs)	1.01 (1.000–1.022)	0.051		
BMI (kg/cm^2^)	1.02 (0.966–1.068)	0.531		
eGFR (ml/min/1.73 m^2^)	0.99 (0.988–1.000)	0.044	0.99 (0.987–1.000)	0.056
First ureteral stone episode (yes)	2.66 (1.948–3.644)	<0.001	2.53 (1.819–3.510)	<0.001
Previous SSP (yes)	2.10 (0.969–4.527)	0.060		
Side (right)	0.97 (0.716–1.318)	0.854		
Location (lower)	2.46 (1.794–3.366)	<0.001	2.43 (1.738–3.390)	<0.001
Stone size (mm)	0.91 (0.850–0.976)	0.008	0.94 (0.872–1.006)	0.071
α-blocker usage (yes)	0.72 (0.474–1.105)	0.134		
Presence of hydronephrosis (yes)	0.30 (0.090–0.997)	0.049	0.44 (0.126–1.515)	0.192
Radiopaque (yes)	2.00 (1.465–2.727)	<0.001	1.91 (1.369–2.655)	<0.001
Multiple stones (yes)	0.94 (0.290–3.013)	0.911		
RBC count in urine (/μL)	1.00 (1.000–1.000)	0.962		
WBC count in urine (/μL)	1.00 (0.999–1.001)	0.940		

BMI, body mass index; CI, confidence interval; eGFR, estimated glomerular filtration rate; RBC, red blood cell count; SSP, spontaneous ureteral stone passage; WBC, white blood cell count.

In the sub-analysis according to stone size (<5 mm), SSP was significantly associated with first ureteral stone episode (OR = 3.26 (2.124–4.999), p<0.001) and location of stone (lower) (OR = 1.93 (1.221–3.035), p = 0.005) (Table 3). Usage of α-blocker (MET) and opacity of stones were not identified as significant predictors of SSP. In the group with stone size 5–10 mm, SSP was significantly associated with the first ureteral stone episode (yes) (OR = 2.53 (1.819–3.810), p<0.001), location of stone (lower) (OR = 2.43 (1.738–3.390), p<0.001), and opacity of stones (radiopaque) (OR = 1.91 (1.369–2.655), p<0.001) in the multivariate analysis (Table 3).

**Table 3:** Univariate and multivariate logistic regression analyses of the factors predicting the spontaneous passage of ureteral stones according to size of stones.

**Stone size <5 mm**	*Univariate*		*Multivariate*	
	Odds Ratio (95% CI)	P-value	Odds Ratio (95% CI)	P-value
Sex (male)	0.97 (0.636–1.490)	0.903		
Age (yrs)	1.00 (0989–1.016)	0.750		
BMI (kg/cm^2^)	1.05 (0.976–1.123)	0.198		
eGFR (ml/min/1.73 m^2^)	0.99 (0.986–1.002)	0.120		
First ureteral stone episode (yes)	3.50 (2.306–5.304)	<0.001	3.26 (2.124–4.999)	<0.001
Previous SSP (yes)	2.17 (0.744–6.348)	0.156		
Side (right)	1.07 (0.719–1.603)	0.729		
Location (lower)	1.93 (1.256–2.962)	0.003	1.93 (1.221–3.035)	0.005
Stone size (mm)	0.99 (0.744–1.313)	0.937		
a-blocker usage (yes)	0.47 (0.275–0.790)	0.005	0.58 (0.332–1.026)	0.061
Presence of hydronephrosis	0.30 (0.069–1.298)	0.107		
Radiopaque (yes)	1.65 (1.099–2.476)	0.016	1.48 (0.964–2.272)	0.073
Multiple stones (yes)	1.13 (0.232–5.512)	0.879		
RBC count in urine (/μL)	1.00 (1.000–1.000)	0.476		
WBC count in urine (/μL)	1.00 (0.999–1.001)	0.688		
**Stone size 5–10 mm**	*Univariate*		*Multivariate*	
	Odds Ratio (95% CI)	P-value	Odds Ratio (95% CI)	P-value
Sex (male)	1.39 (0.813–2.361)	0.230		
Age (yrs)	1.03 (1.009–1.049)	0.004	1.04 (1.013–1.058)	0.001
BMI (kg/cm^2^)	0.98 (0.910–1.057)	0.619		
eGFR (ml/min/1.73 m^2^)	0.99 (0.982–1.002)	0.128		
First ureteral stone episode (yes)	1.99 (1.210–3.283)	0.007	2.00 (1.145–3.479)	0.015
Previous SSP (yes)	1.96 (0.635–6.023)	0.242		
Side (right)	0.76 (0.464–1.252)	0.283		
Location (lower)	3.45 (2.06–5.799)	<0.001	3.64 (2.079–6.364)	<0.001
Stone size (mm)	0.86 (0.691–1.060)	0.153		
a-blocker usage (yes)	1.47 (0.687–3.158)	0.319		
Presence of hydronephrosis (yes)	0.35 (0.041–2.947)	0.334		
Radiopaque (yes)	3.20 (1.915–5.339)	<0.001	3.43 (1.956–6.003)	<0.001
Multiple stones (yes)	1.41 (0.145–13.722)	0.768		
RBC count in urine (/μL)	1.00 (1.000–1.000)	0.771		
WBC count in urine (/μL)	1.00 (0.997–1.001)	0.219		

BMI, body mass index; CI, confidence interval; eGFR, estimated glomerular filtration rate; RBC, red blood cell count; SSP, spontaneous ureteral stone passage; WBC, white blood cell count.

**Fig 2:**
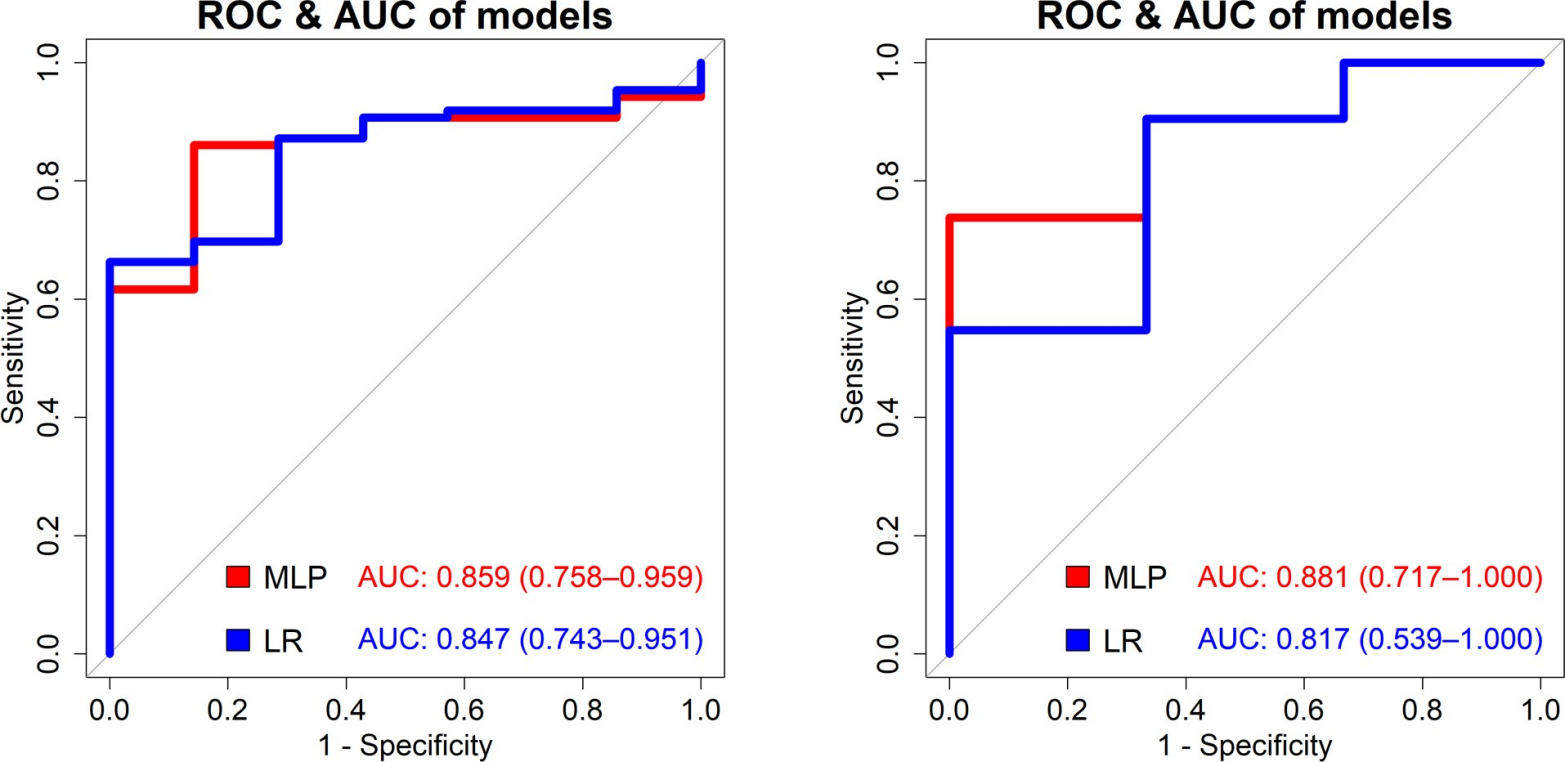
Receiver operating characteristic (ROC) curves for the prediction of the spontaneous passage of ureteral stones. (A) Stone size <5 mm, multilayer perceptron (MLP; area under the curve [AUC] = 0.859) and logistic regression (LR) model (AUC = 0.847). (B) Stone size 5–10 mm, MLP (AUC = 0.881) and LR model (AUC = 0.817).

The predictive performances according to predictive modes are shown in Table 4. For stone size <5 mm, AUCs for MLP and logistic regression were 0.859 and 0.847 (p = 0.410), respectively (Fig 2), and for stone size 5–10 mm, these were 0.881 and 0.817 (p = 0.170), respectively (Fig 2). The sensitivity and specificity of each model are listed in Table 4.

**Table 4:** Comparison between machine learning and logistic regression models for predicting the spontaneous passage of ureteral stones.

**Stone size < 5 mm**	**AUC**	**Sensitivity**	**Specificity**
*Machine learning model (MLP)*	0.859	86.0%	85.7%
*Logistic regression model*	0.847	87.2%	71.4%
**Stone size 5–10 mm**	**AUC**	**Sensitivity**	**Specificity**
*Machine learning model (MLP)*	0.881	71.4%	100.0%
*Logistic regression model*	0.817	90.5%	66.7%

AUC area under the receiver operating curve, MLP multilayer perceptron.

Optimal cut-off was considered the point closest to the top-left part of the plot.

## Discussion

The greatest dilemma the urologist faces today is to decide whether to wait for SSP or immediately perform intervention for ureterolithiasis. Of the parameters for diagnostic evaluation recommended by the guidelines [9], in addition to the widely renowned predictors for SSP, location and opacity of the stones and the first ureteral stone episode were identified as the predictors of SSP in this study. In addition to analysis using logistic regression models, this study investigated the predictability of machine learning based prediction models using MLP, a class of deep learning method. To the best of our knowledge, no report has compared the predictability of deep learning or machine learning with conventional approaches.

According to the current literature, the size and location of the stone, serum C-reactive protein (CRP) concentration, neutrophil to lymphocyte ratio, pyuria, hydronephrosis, helical CT findings of perinephric fat stranding and the tissue-rim sign related to inflammatory changes, and Hounsfield unit of stone are predictors associated with SSP [10–14]. However, if no intervention is planned, an examination of sodium, potassium, CRP, and blood coagulation time may be omitted [9]. Measuring Hounsfield units was recommended to decide whether to consider ESWL. There were no definite criteria, such as perinephric fat stranding and tissue-rim sign, to classify the findings in CT. Therefore, we did not include these variables as potential predictors in our analysis. Additionally, our team is currently investigating the predictors for SSP that includes image analysis of Hounsfield units, anatomical abnormalities, and malformation.

Stone size and location are generally considered the most important factors associated with the possibility of SSP. Limited data were found on SSP according to stone size. Ueno et al. analyzed 520 ureteral calculi based on size and reported that 286 (55.0%) calculi passed spontaneously, and the mean length was 6.3 mm [15]. In our previous study, we reported an SSP rate of 88.2% for stones up to 5 mm, and 62.2% for 5–10 mm stones [10]. However, a meta-analysis of five patient groups (224 patients) estimated that 68% of stones ≤5 mm would pass spontaneously (95% CI: 46% to 85%). For stones >5 mm and ≤10 mm, a meta-analysis of three groups (104 patients) estimated that 47% would pass spontaneously (95% CI: 36% to 59%). This study showed that the SSP rates within 4 weeks for patients with stones <5 mm, and those with stones 5–10 mm, were 75.6% and 68.2%, respectively [4]. Our findings, which are from the largest sample of 833 patients, were in accordance with the previous studies.

The parameters, including a history of ureteral stones, α-blocker usage (MET), and hydronephrosis, were presented as predictive factors for SSP in other previous studies. Ozcan et al. presented a history of SSP as the positive predictive factor for SSP in 251 patients with 4–10 mm distal ureteral stones [16]. However, the previous SSP might have caused permanent changes in the ureter owing to inflammation. In our cohort, a history of SSP was not found to be a significant parameter in the univariate analysis. The first ureteral stone episode was found to be a positive predictor of SSP in the multivariate analysis. Patients using α-blockers (MET), calcium channel blockers, and phosphodiesterase type 5 inhibitors were more likely to pass stones with fewer colic episodes than those not undergoing such therapy [17–19]. The EAU guideline strongly recommends α-blockers as MET for (distal) ureteral stones **>** 5 mm [9]. However, in our study population, MET was not a significant predictor of SSP. Further, in Korea, medication for MET is not reimbursed for patients with ureteral stones; thus, only limited patients have received medication for MET based on the physician’s preference. The presence of hydronephrosis was a negative predictor of SSP in a previous study [20]. However, in our study, hydronephrosis was not found to be a significant parameter in multivariate analysis. We presume that this is because the degree of hydronephrosis could be affected by the time that CT was performed and other physical parameters related to stones, such as shape, size and anatomical features in the ureter.

According to EAU guidelines, an exact cut-off size for stones that may pass spontaneously cannot be provided; however, ureteral stones <6 mm can pass spontaneously in well-controlled patients [9]. However, for stones measuring 5–10 mm, the optimal treatment strategy remains unclear. Therefore, clinicians may prefer interventions, such as ESWL or ureteroscopic ureterolithotomy, to conservative management.

Although each medical evaluation and parameter have their own meaning and roles for evaluation of the disease status of patients, they could not accurately predict patient outcome. Prediction of patient outcome in the medical fields including the fields of ureterolithiasis, requires incorporation of several parameters and handling of complex and big data. We believe that is the reason why models are being developed to predict patient outcome including the fields of ureterolithiasis. We attempted to develop predicting models using artificial intelligence (AI) technology. Indeed, the number of articles applying machine learning to medical research has been growing rapidly in recent years [21, 22]. In particular, deep learning, a class of machine learning, is increasingly being applied in the field of diagnosis and prediction related to medical imaging, yielding impressive results [8]. It will benefit both patients and clinicians who are willing to adopt modern technologies.

In the fields of ureterolithiasis, AI has been widely used in predicting stone-free rate such as the recent study by Shabaniyan et al. reporting 94.8% accuracy in predicting postoperative outcome of a percutaneous nephrolithotomy [23]. However, to the best of our knowledge, no study has been reported for predicting SSP by AI techniques [2]. While this study implemented a deep learning approach for constructing the SSP prediction model, the “black box” nature, or incapability to identify the reason of each decision, is a limitation of AI based diagnosis [22]. The doctors will rarely follow the advice of a machine if they cannot see the reasoning underlying that advice, especially when the responsibility for the patient will remain with the clinicians [22, 24]. There are ongoing studies on this, and some of them are showing achievements in this interpretability.

Our study had several strengths. Most previous studies only described the predicting factors of SSP [11, 14, 25, 26]. To our knowledge, this is the first study to provide prediction models of SSP based on the diagnostic parameters recommended by the guidelines, with the largest cohort of its kind. Moreover, our study compared the deep learning method, MLP based on Keras framework, with the conventional statistic method, logistic regression in predicting SSP. Although MLP has higher AUC and accuracy than logistic regression, there were no statistical differences in the predictive power between the two models. However, MLP showed a higher specificity than logistic regression, especially for ureteral stones 5–10 mm in size. This grey zone is where clinicians have difficulty in deciding between ureterolithiasis or wait for SSP. Since MLP showed 100% specificity in predicting SSP for ureteral stones of 5-10mm in size, we believe that we could utilize MLP in real clinical settings to suggest wait for SSP in patients with 5-10mm ureteral stones.

However, there were several limitations. First, the level of patient compliance in terms of fluid intake would have differed among patients; hence, this could not be included as a parameter. Second, although radiopaque stone was identified as a significant predictor of SSP, we have not analyzed parameters in images, including using the Hounsfield unit. Third, although the original intent for this study was not focused on finding minimal-optimal subset of features, we could investigate on minimal-optimal features of SSP by using AI techniques such as maximum relevance-minimum redundancy algorithm or linear discriminant analysis for dimensionality reduction methods in our future study.

In this study, predictive models of SSP were developed in patients with unilateral ureteral stones. In particular, our findings can be useful in identifying low likelihood of SSP for 5–10 mm size ureteral stones with no definite treatment guidelines. Although the deep learning method MLP based on Keras framework did not show superior performance in predicting SSP compared to the conventional statistical method, logistic regression, future studies should attempt to improve the predictive power using image analysis.

## Supporting information

S1 Data (XLSX)
